# Influence of Initial Severity of Depression on the Effectiveness of a Multimodal Therapy on Depressive Score, Heart Rate Variability, and Hemodynamic Parameters

**DOI:** 10.3390/ijerph19169836

**Published:** 2022-08-10

**Authors:** Sascha Ketelhut, Emanuel Wehlan, Gerhart Bayer, Reinhard G. Ketelhut

**Affiliations:** 1 Institute of Sport Science, University of Bern, 3012 Bern, Switzerland; 2 Cardiology and Sports Medicine, Medical Center Berlin (MCB), 10559 Berlin, Germany; 3 Institute of Sport Science, Humboldt University of Berlin, 10115 Berlin, Germany

**Keywords:** depression, pretreatment severity of depression, multimodal therapy, cardiovascular risk, blood pressure, heart rate variability

## Abstract

Depression is a major cause of disability among populations worldwide. Apart from primary symptoms, depressed patients often have a higher cardiovascular risk profile. Multimodal therapy concepts, including exercise, have emerged as promising approaches that not only improve depressive symptoms but also have a positive impact on cardiovascular risk profile. However, controversies have arisen concerning the influence of baseline severity on the effects of therapy concepts for this demographic. This study assessed whether pretreatment severity moderates psychological and physiological treatment outcomes of a multimodal therapy. A total of 16 patients diagnosed with mild depression (MD) and 14 patients diagnosed with severe depression (SD) took part in a 3-month outpatient multimodal treatment therapy. Before and after the treatment, depression score (Beck Depression Inventory (BDI)), peripheral systolic (pSBP) and diastolic (pDBP) blood pressure, central systolic (cSBP) and diastolic (cDBP) blood pressure, pulse wave velocity (PWV), heart rate (HR), and parasympathetic parameters of heart rate variability (RMSSD) were assessed. Significant time effects were detected for BDI (−20.0 ± 11.6, *p* > 0.001, η^2^ = 0.871), pSBP (−4.7 ± 6.8 mmHg, *p* < 0.001, η^2^ = 0.322), pDBP (−3.5 ± 6.9 mmHg, *p* = 0.01, η^2^ = 0.209), cSBP (−4.8 ± 6.5 mmHg, *p* < 0.001, η^2^ = 0.355), cDBP (−3.6 ± 6.8 mmHg, *p* = 0.008, η^2^ = 0.226), PWV (−0.13 ± 0.23 m/s, *p* = 0.008, η^2^ = 0.229), HR (4.3 ± 8.8 min^−1^, *p* = 0.015, η^2^ = 0.193), RMSSD (−12.2 ± 23.9 ms, *p* = 0.017, η^2^ = 0.251), and and SDNN (10.5 ± 17.8 ms, *p* = 0.005, η^2^ = 0.330). Significant time × group interaction could be revealed for BDI (*p* < 0.001, η^2^ = 0.543), with patients suffering from SD showing stronger reductions. Pretreatment severity of depression has an impact on the effectiveness of a multimodal therapy regarding psychological but not physiological outcomes.

## 1. Introduction

Depression is a highly prevalent disorder, with approximately 280 million people worldwide suffering from major depression [1]. Depression is a debilitating condition resulting in persistent feelings of sadness, loss of pleasure or interest, and decreased motivation, often leading to a reduced quality of life and premature mortality [2].

Additionally, depression is associated with various physical comorbidities such as cardiovascular diseases [3]. According to the literature, depression increases the risk of hypertension [4], myocardial infarction, and coronary heart disease [5,6]. Furthermore, depression and anxiety scores are positively associated with systolic blood pressure (BP) independent of lifestyle confounders [7]. Kampling et al. [8] reported that the prevalence of major depression in patients diagnosed with type 1 diabetes is twice as high as in the reference population. According to Ladwig et al. [9], depression represents a similar risk factor for cardiovascular disease as overweight or metabolic disorders. A recent meta-analysis identified cardiovascular diseases as the leading cause of premature mortality amongst the depressed [3].

Given the high prevalence and personal burden, there is an urgent need to improve treatment approaches for depression. Standard treatment options include pharmacotherapy and psychological interventions such as cognitive behavior therapy (CBT) and interpersonal therapy. However, exercise as an adjunct to conventional therapies is gaining attention. It is widely acknowledged that regular physical activity or exercise can reduce the risk of developing major depression [10,11,12,13]. Apart from the preventive effect, a growing body of literature suggests that exercise is an effective strategy for treating depression, reducing depressive symptoms, and improving quality of life [14,15,16]. Previous research suggests that exercise training can induce similar effects as psychological or pharmacological treatments [17,18,19,20]. However, the number of studies reporting these comparisons is still small [17]. Additionally, exercise can not only alleviate depression but also benefit other health outcomes such as cardiovascular risk factors [21]. Regular exercise is considered a major lifestyle approach for the treatment and prevention of hypertension [22]. Apart from peripheral BP, other early markers for future cardiovascular morbidity and mortality such as central BP, pulse wave velocity (PWV), and heart rate variability (HRV), are also positively affected by regular exercise [23,24].

Although the effects of both psychological interventions and exercise are widely acknowledged, there is little evidence on the moderators of treatment effects in depressive patients. It remains questionable whether initial depression severity moderates the effectiveness of various interventions, thus making specific treatment approaches more suitable for specific patients. Previous studies suggest that patients who are more severely depressed at baseline demonstrate more significant treatment effects than those who are less severely depressed [25,26]. On the contrary, Lovell et al. [27] discovered a greater benefit in less severely ill patients. According to two meta-analyses by Furukawa et al. [28] and Bower et al. [29], baseline depression severity did not influence symptom change after low-intensity interventions and CBT.

Unfortunately, there is relatively little rigorous evidence on how pretreatment severity moderates the efficacy of multimodal therapy, with an emphasis on exercise. It is not clear if such a treatment approach is suitable for patients with different depression levels. Furthermore, no previous study has assessed if depression severity influences treatment effects regarding relevant comorbidities like cardiovascular risk factors.

Therefore, this study assessed whether more severely depressed patients show better or worse treatment effects on depression scores and different cardiovascular risk factors, such as peripheral and central BP, PWV, and HRV, after multimodal therapy emphasizing exercise compared to less ill patients.

## 2. Materials and Methods

### 2.1. Study Design and Participants

An a priori power analysis was conducted utilizing G*Power (Version 3.1.2; Heinrich Heine Universität, Dusseldorf, Germany). Assuming an effect size of 0.8 with an alpha level of 0.05 using a group effect on change in depression score as the primary outcome measure, 26 participants were required for the study to have sufficient power. Thirty patients (48 ± 11 years, body mass index (BMI) 25.0 ± 3.4 kg/m^2^, 60% women) from a psychosomatic day clinic participated in the study. Patients were eligible for the study if they (1) provided written informed consent, (2) had no physical limitations to exercise, (3) had been diagnosed with depressive symptoms, (4) were not taking antidepressants, and (5) were 18 years or older. Before participation, patients underwent a medical screening by a physician and a psychotherapist. Based on their depression scores, the participants were classified as either mildly depressed (MD) (Beck Depression Inventory (BDI) < 20) or severely depressed (SD) (BDI > 29) [30].

All participants received a verbal and written explanation of the study’s objective and experimental procedures and provided written informed consent. The study was conducted in accordance with the Helsinki Declaration and approved by the Research Ethics Board of the Medical Center Berlin (Medical Center Berlin (MCB), Berlin, Germany, EA20170208-4). After a baseline examination, all participants took part in a 3-month outpatient psychosomatic therapy.

### 2.2. Measurements

Before and after the intervention, different outcomes were assessed. All measurements were performed on the same weekday in the morning hours after overnight fasting in a temperature-controlled room (23 ± 1 °C). Subjects were instructed to refrain from consuming caffeinated, alcoholic beverages, or nicotine four hours before the examination. In addition, the subjects were instructed to avoid any intense physical activity for 48 h prior to the study. Five subjects reported being on BP medication. In consultation with their physicians, the medication was not changed throughout the intervention. All measurements were conducted at the psychosomatic day clinic using the same equipment and procedure under standardized conditions.

#### 2.2.1. Hemodynamic Measurements

Hemodynamic parameters (peripheral and central BP, PWV) were determined non-invasively using Mobil-O-Graph^®^ (PWA-Monitor, IEM, Stollberg, Germany), which is a clinically validated device for hemodynamic measurements [31]. After a 10 min supine rest, three readings were performed on the right upper arm using customized arm cuffs. The arm was placed on an armrest to ensure that the heart and pressure cuff were at the same level. All hemodynamic measurements were conducted by the same study staff member with the same device before and after the intervention. The average of the second and third readings was used for analysis.

#### 2.2.2. Cardiac Autonomic Function

The HRV was obtained using a heart rate monitor and a chest strap (Polar RS800 CX^®^, Polar Electro OY, Kempele, Finland). After a 5 min supine rest period and a stabilized HRV signal, a 10 min measurement was conducted. A sampling rate of 1000 Hz was used to record the RR intervals [32]. Throughout the measurements, patients were instructed to breathe normally, not speak, and stay calm.

HRV analysis was performed on the data collected from the last 5 min of the measurement. The raw data was processed using the software “Kubios HRV” version 2.1 (Biosignal Analysis and Medical Imaging Group, Department of Physics, University of Kuopio, Kuopio, Finland). Kubios preprocessing settings were at the default values, including the RR detrending method, which was kept at “Smoothen priors” (Lambda = 500) [33]. Only data with an error ratio below 5% were considered [32,34]. The root mean square of successive differences between normal heartbeats (RMSSD in ms), and the standard deviation of all normal-to-normal intervals (SDNN) was analyzed.

#### 2.2.3. Depression SCORE

Depression score was measured using the BDI. The BDI self-reporting inventory for evaluating the severity of depression in normal and psychiatric populations [35]. The internal consistency for the BDI ranges from 0.73 to 0.92, with a mean of 0.86 [36]. Scoring is achieved by adding up the ratings for all 21 items, with 0 being the minimum and 63 being the maximum score. Higher scores indicate greater symptom severity. In those diagnosed with depression, scores of 0–13 indicate minimal depression, 14–19 (mild depression), 20–28 (moderate depression), and 29–63 (severe depression) [30]. The 21 groups of statements consist of the symptoms: “dysphoria, pessimism, failure, loss of pleasure, feelings of guilt, punishment, self-denial, self-criticism, suicidal thoughts, crying restlessness, loss of interest, determination, worthlessness, energy loss, sleep disorders, irritability, loss of appetite, difficulty concentrating, fatigue and loss of libido” [37].

### 2.3. Multimodal Psychosomatic Therapy

The patients visited a psychosomatic day clinic each day (5 days/week) from 9:00 am to 5:00 pm. The multimodal psychosomatic therapy comprised individual and group CBT, stress regulation training, dance and music therapy, art therapy, acupuncture, nutritional counseling, and meditation. Additionally, Yoga and Qi Gong were performed 1 × 60 min/week each. Furthermore, an exercise program was performed three times per week (2 × 45 min and 1 × 60 min per week). Alle therapeutic offers were conducted by qualified clinicians (CBT, acupuncture), therapists (art, dance and music, nutritional counseling, stress regulation, meditation), or trained specialists (Yoga, Qi Gong, exercise program).

The exercise intervention consisted of varied group-based exercises, including strengthening exercises, bodyweight workouts, endurance exercises, coordination tasks, balance training, stretching, and small games. The goal was to offer a variety of tasks and activities during each session that ensure a high level of active movement time and are experienced as enjoyable.

### 2.4. Statistics

Statistical analyses were performed using SPSS version 27 (IBM, Chicago, IL, USA). The analyses were based on the intention-to-treat principle. Data normality was assessed on each variable using a histogram and the Kolmogorov–Smirnoff test. Differences in subject characteristics between the groups at baseline were determined using an independent samples *t*-test for continuous variables and Chi-square tests for categorical variables. A Levene test was used to verify the homogeneity of variance. ANOVAs were performed to determine within-group effects. A series of two-way (groups: MD vs. SD) ANOVAs with repeated measures (time: baseline versus post-intervention) were performed to determine time × group interactions. Post hoc analyses with Bonferroni’s correction were performed if necessary. The effect size was measured by Partial Eta Square (η^2^). In the present study, small, medium, and large effect sizes were designated as 0.01 ≤ 0.06, 0.06 < 0.14, and ≥0.14, respectively [38].

## 3. Results

The session attendance rates for the therapeutic sessions were 91% for the MD and 92% for the SD. No adverse events were documented in any of the patients during the intervention period. Patients’ characteristics are summarized in Table 1. According to the BMI, eight patients were classified as overweight and 6 as obese [39]. With regard to the waist-to-height ratio, 14 of the included patients showed values in the overweight range. According to the BP classification of the European Society of Cardiology (ESC) [40], eight patients had a high normal BP, and eleven participants revealed BP in the hypertensive range. Apart from the BDI scores, there were no significant differences between the MD and the SD.

Significant time effects were detected for BDI (−20.0 ± 11.6, *p* > 0.001, η^2^ = 0.871), pSBP (−4.7 ± 6.8 mmHg, *p* < 0.001, η^2^ = 0.322), pDBP (−3.5 ± 6.9 mmHg, *p* = 0.01, η^2^ = 0.209), cSBP (−4.8 ± 6.5 mmHg, *p* < 0.001, η^2^ = 0.355), cDBP (−3.6 ± 6.8 mmHg, *p* = 0.008, η^2^ = 0.226), PWV (−0.13 ± 0.23 m/s, *p* = 0.008, η^2^ = 0.229), heart rate (HR) (4.3 ± 8.8 min^−1^, *p* = 0.015, η^2^ = 0.193), RMSSD (12.2 ± 23.9 ms, *p* = 0.017, η^2^ = 0.251), and SDNN (10.5 ± 17.8 ms, *p* = 0.005, η^2^ = 0.330). No significant time effects were detected in BMI (0.14 ± 0.61, *p* = 0.236, η^2^ = 0.05) and WHtR (−0.002 ± 0.009, *p* = 0.306, η^2^ = 0.037). Significant time × group interaction could be revealed for BDI, with patients suffering from SD showing stronger reductions (−11.6 ± 4.9 vs. −28.4 ± 10.1, *p* < 0.001, η^2^ = 0.543) (Table 2).

**Figure 1:**
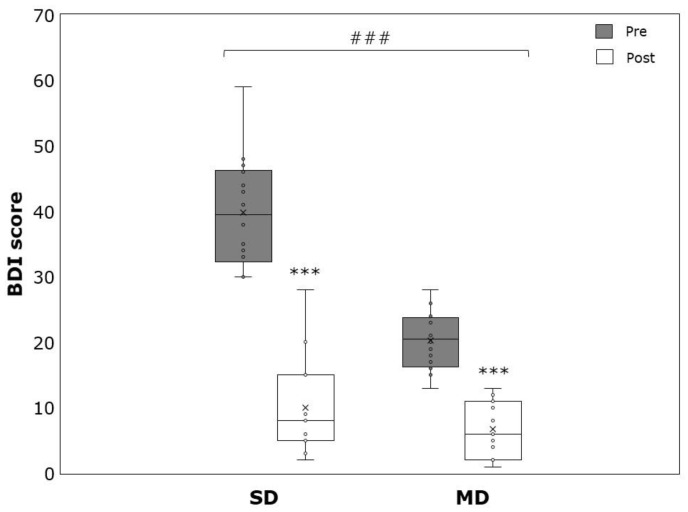
Beck Depression Inventory (BDI) score before (pre) and after (post) the intervention period for severely depressed (SD) and mildly depressed (MD). *** *p* < 0.001 within-group difference. ### *p* < 0.001 time × group effects.

**Figure 2:**
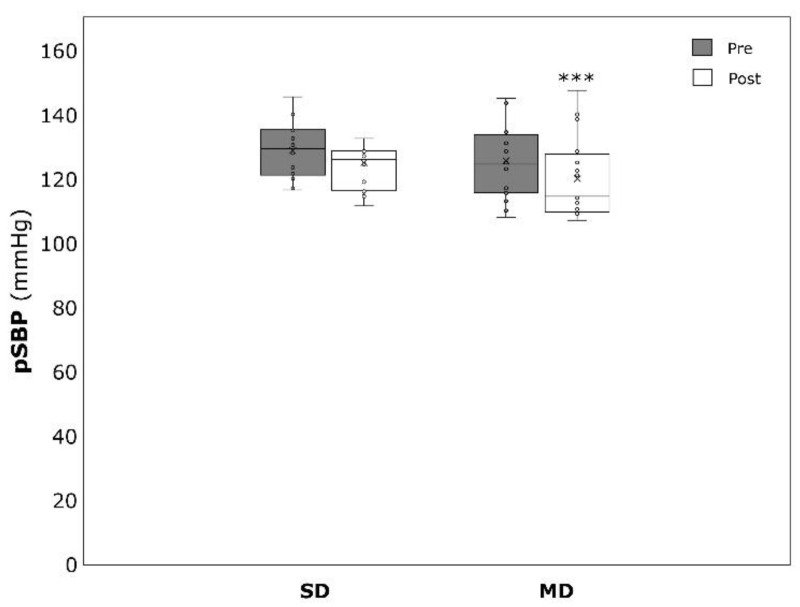
Peripheral systolic blood pressure (pSBP) before (pre) and after (post) the intervention period for severely depressed (SD) and mildly depressed (MD). *** *p* < 0.001 within-group difference.

**Figure 3:**
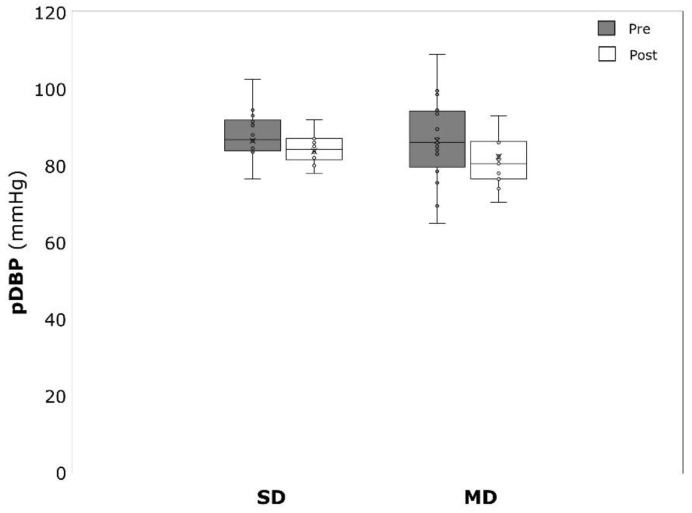
Peripheral diastolic blood pressure (pDBP) before (pre) and after (post) the intervention period for severely depressed (SD) and mildly depressed (MD).

Significant within-group effects could be detected for BDI amongst the MD (−11.6 ± 4.9, *p* < 0.001, η^2^ = 0.859) and the SD (−28.4 ± 10.1, *p* > 0.001, η^2^ = 0.894) patients (Figure 1). Concerning pSBP, only the MD revealed significant changes over time (−5.6 ± 5.5 mmHg, *p* < 0.001, η^2^ = 0.525). No significant changes could be detected for pSBP in the SD (−3.7 ± 8.1 mmHg, *p* = 0.109, η^2^ = 0.185) and for pDBP in the SD (−2.8 ± 5.1 mmHg, *p* = 0.061, η^2^ = 0.245) and the MD (−4.2 ± 8.3 mmHg, *p* = 0.063, η^2^ = 0.212) (Figure 2 and Figure 3).

**Figure 4:**
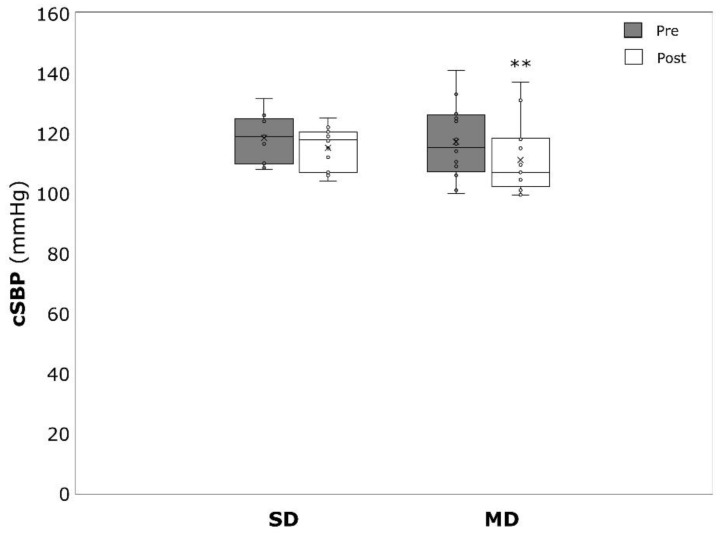
Central systolic blood pressure (pSBP) before (pre) and after (post) the intervention period for severely depressed (SD) and mildly depressed (MD). ** *p* < 0.01 within-group difference.

**Figure 5:**
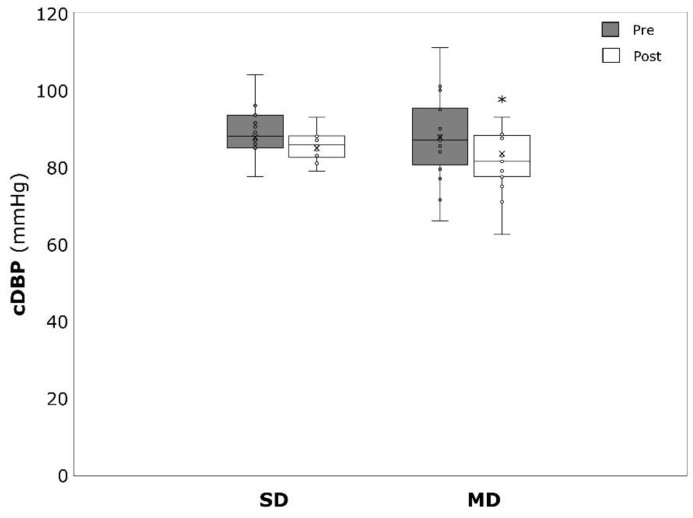
Central diastolic blood pressure (cDBP) before (pre) and after (post) the intervention period for severely depressed (SD) and mildly depressed (MD). * *p* < 0.05 within-group difference.

cSBP significantly decreased in the MD (−6.1 ± 6.6 mmHg, *p* = 0.002, η^2^ = 0.476) but not in the SD (−3.3 ± 6.4 mmHg, *p* = 0.080, η^2^ = 0.217) (Figure 4). Similar results could be detected for cDBP which was significantly reduced amongst the MD (−4.3 ± 7.9, *p* = 0.046, η^2^ = 0.240) but not the SD (−2.9 ± 5.3 mmHg, *p* = 0.066, η^2^ = 0.236) (Figure 5).

**Figure 6:**
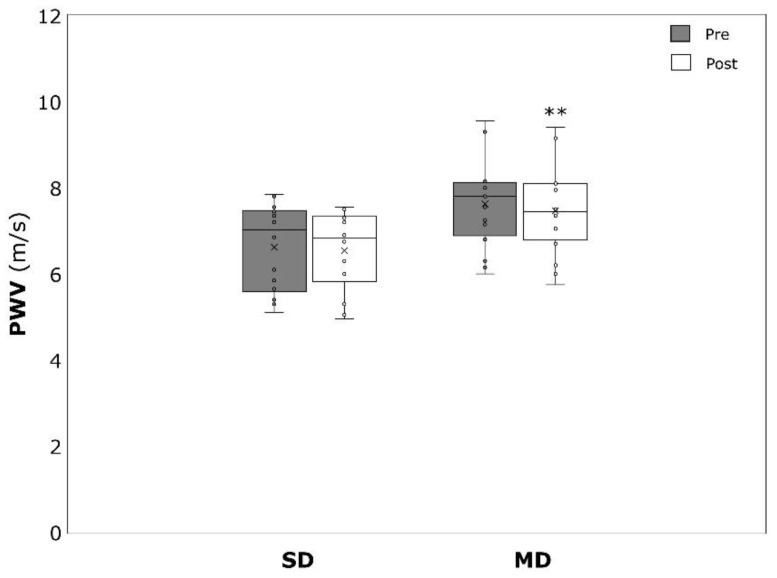
Pulse wave velocity (PWV) pressure before (pre) and after (post) the intervention period for severely depressed (SD) and mildly depressed (MD). ** *p* < 0.01 within-group difference.

**Figure 7:**
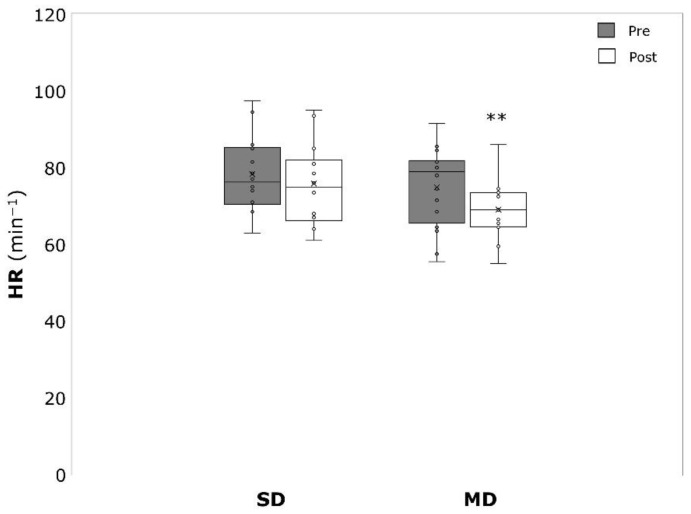
Heart rate (HR) before (pre) and after (post) the intervention period for severely depressed (SD) and mildly depressed (MD). ** *p* < 0.01 within-group difference.

**Figure 8:**
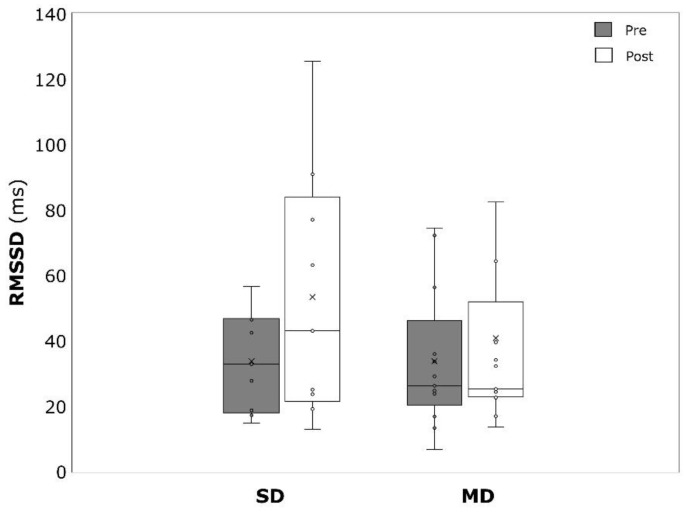
Root mean square of successive differences between normal heartbeats (RMSSD) before (pre) and after (post) the intervention period for severely depressed (SD) and mildly depressed (MD).

**Figure 9:**
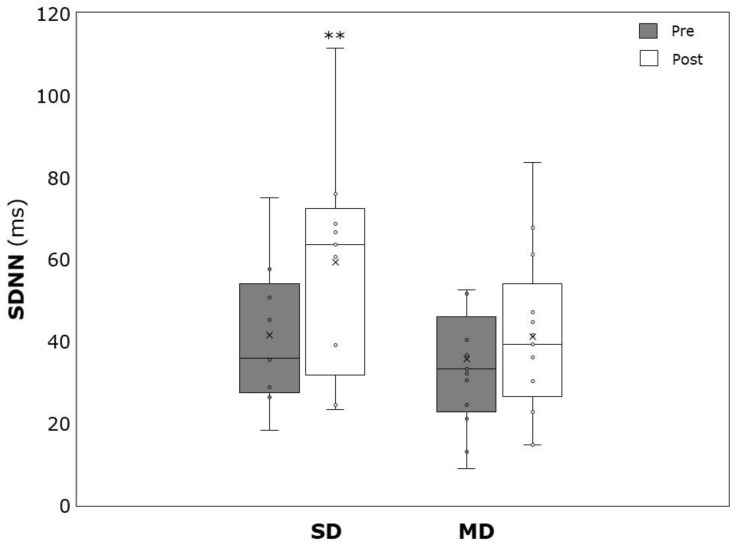
Standard deviation of all normal-to-normal intervals before (SDNN) (pre) and after (post) the intervention period for severely depressed (SD) and mildly depressed (MD). ** *p* < 0.01 within-group difference.

PWV significantly decreased over time in the MD (−0.17 ± 0.19 m/s, *p* = 0.004, η^2^ = 0.431), but not the SD (−0.08 ± 0.27 m/s, *p* = 0.280, η^2^ = 0.089) (Figure 6). The same was true for HR, which was significantly lower after the intervention in the MD (−5.8 ± 7.4 min^−1^, *p* = 0.007, η^2^ = 0.397) but not in the SD (−2.5 ± 10.1 min^−1^, *p* = 0.377, η^2^ = 0.061) (Figure 7). RMSSD was not affected by the intervention in both SD (19.6 ± 27.6 ms, *p* = 0.066, η^2^ = 0.362) and MD (7.0 ± 20.7 ms, *p* = 0.243, η^2^ = 0.111) (Figure 8). SDNN significantly increased in the SD (17.8 ± 19.8 ms, *p* = 0.027, η^2^ = 0.477) but not in the MD (5.4 ± 14.9 ms, *p* = 0.218, η^2^ = 0.124) (Figure 9).

## 4. Discussion

This is the first trial to examine how pretreatment severity of depression moderates the effectiveness of a multimodal therapy, with an emphasis on exercise regarding depressive symptoms and hemodynamic parameters. The findings suggest that the intervention improved depression scores and different cardiovascular parameters. Patients with a higher initial depression score showed greater improvements in depressive symptoms throughout the intervention. Baseline depression scores did not moderate changes in cardiovascular parameters.

The detected effects on depression scores are in accordance with previous literature. A meta-analysis including 2470 patients participating in low-intensity interventions reported a significant interaction between baseline severity and treatment effect, suggesting that patients who are initially more depressed demonstrate greater treatment effects [29]. On the contrary, Cuijpers et al. [41] found no association between the study’s effect size and mean baseline depression severity in their meta-analysis on the effects of different psychotherapies. This is in accordance with Furukawa and colleagues [28], who reported no statistically significant influence of baseline depression severity on subsequent differential symptom change after CBT. Similarly, Driessen et al. [25] found no effect of pre-treatment depression scores on the effect size of different psychotherapies. However, the scholars did indicate that the effect size was greater and statistically significant in high-severity patients and smaller and non-significant in low-severity patients.

Although the literature is equivocal on the effects of pretreatment severity, the results of this study add to the body of literature confirming that pretreatment depression levels moderate posttreatment effect size. However, previous studies only examined low-intensity interventions, CBT, or different psychotherapies. Intervention approaches applying an intensive multimodal therapy concept with an emphasis on exercise have not been studied.

Scientific evidence regarding increased cardiovascular risk among depressed patients underlines the growing need to develop successful treatments for depression that aim to reduce its impact on cardiovascular parameters [42]. Apart from the positive effects on depression scores, the multimodal therapy concept had a positive effect on cardiovascular risk makers such as peripheral and central BP, PWV, and HRV.

The effects on peripheral BP are consistent with previous studies indicating the positive effects of various psychotherapeutic treatments [43,44,45]. Although the reduction in peripheral BP was only moderate (5/4 mmHg), it is well recognized that already modest reductions in BP are associated with a significantly lower risk for cardiovascular complications [46].

Emerging evidence now suggests that besides peripheral BP, other hemodynamic parameters such as central BP and PWV are more strongly associated with preclinical organ damage [47] and better associated with future cardiovascular events [48]. Data from a prospective study indicates that PWV is associated with a decline in endothelial function and is a precursor for future cardiovascular risk, even after accounting for other established risk factors [49]. Regular aerobic exercise has been shown to be effective in reducing arterial stiffness [50]. Although previous studies report an association between depressive symptoms and PWV [51,52], the effects of CBT or other psychotherapeutic treatments have not been assessed.

Apart from hemodynamic effects, the intervention led to reductions in HR and improvements in HRV. HRV measures the beat-to-beat fluctuation in the time intervals between adjacent heartbeats, providing a sensitive measurement of cardiac autonomic control [53]. HRV is increasingly being used to quantify the heart’s autonomic regulation, allowing for the identification of patients at increased risk for cardiac events [54]. According to a vast body of literature, HRV is positively correlated with depression [55,56]. Unfortunately, traditional interventions for depression, such as antidepressants and psychotherapy, are not able to improve HRV, even after successfully reducing depressive symptoms [57,58]. However, approaches including HRV biofeedback have shown similar effects on HRV amongst the depressed [59,60], as seen in the present study. Although no time × group interactions were detected, a significant increase in SDNN was evident in the SD.

Interestingly, there were no differences in hemodynamic parameters between MD and SD at baseline. This contradicts previous studies reporting a higher BP amongst patients with higher depression scores [7]. Furthermore, we detected no difference between the groups regarding the changes in hemodynamic parameters. We expected that the SD, which presented a more substantial reduction in depression score, would show greater improvements in hemodynamic parameters. This assumption stems from the fact that depression is associated with different physiological alterations that directly affect the cardiovascular system. According to literature, depression is associated with increased markers of inflammation [61], hypercortisolism, impaired HRV, elevated catecholamines, endothelial dysfunction [62], platelet function abnormalities [63], and impaired fibrinolysis [64], all of which modulate hemodynamic parameters [65]. We expected that the stronger reduction in depression score in the SD would translate into more pronounced physiological adaptations, resulting in more significant changes in respective risk markers. However, this does not seem to be the case. Unfortunately, the evidence available from this study does not clarify the underlying physiological mechanisms that mediate the changes in the hemodynamic parameters. Thus, we cannot draw any conclusion on the reason for the similar effects. We can only speculate that the high amount of physical exercise (Yoga, Qi Gong, exercise program) was the primary modulator for the changes in hemodynamic parameters and that the psychological condition had less impact.

In summary, the results show that cardiovascular risk factors are treatable with psychotherapeutic interventions aimed at reducing the symptoms of depression. By applying a multimodal treatment approach for depression, it is possible to improve cardiovascular risk profile to a significant degree, irrespective if patients suffer from severe or moderate depression. Based on the increasing prevalence and the mounting economic burden of both depression and cardiovascular diseases, more research on intervention approaches targeting both diseases is needed.

### Limitations

There are some methodological limitations that warrant discussion. First, we used self-rating scales (BDI) to determine depression severity in patients. Observer-rating scales are more sensitive to change than self-rating scales [66]. Second, the sample size was relatively small as the group size of the therapy was restricted. The present findings require replication with a larger sample to test the interaction.

Third, the study lacks a non-treatment control group, as this was rejected for ethical reasons. Thus, it is not clear whether the changes observed over time and the time × group interactions are due to treatment alone. According to a meta-analysis addressing the untreated short-term course of major depression, depressed subjects randomized to the control groups reported a mean reduction in symptoms of 10–15% [67]. Although this spontaneous response is relatively high, the changes in BDI scores in the present study were significantly higher (28%). Even if the natural course of depression could have influenced the results of the present study, it is unlikely to account for the full extent of the changes observed. Since the authors did not report differences in rates of spontaneous improvement as a function of depression severity [67], it is not expected that there was an effect on the time × group interactions.

Fourth, due to the multimodal nature of the intervention approach, we are not able to determine the role of each part of the intervention alone. However, it was not the goal to assess which approach is the most effective but rather to determine the multimodal intervention as a whole.

Fifth, we only obtained short-term HRV readings. An earlier study shows that HRV analyses from short-term recordings seem less reproducible than long-term readings [68]. Lastly, participants were highly selected and recruited from one study center. Further multicenter studies are highly warranted.

Sixth, no follow-up assessments were conducted, thus it is not clear how long the effects will last. However, previous research suggests that the physiological and psychological effects of different treatment approaches for depression can persist for at least a few months [17,43,59,69].

## 5. Conclusions

In conclusion, the present findings are the first to illustrate that a multimodal therapy concept with an emphasis on exercise induces beneficial effects on depression scores and different cardiovascular parameters. Furthermore, patients with a higher initial depression score showed more significant improvements in depressive symptoms throughout the intervention. Changes in cardiovascular parameters were not moderated by baseline depression score.

## Figures and Tables

**Table 1:** Baseline demographic characteristics of patients (means (M) ± standard deviations (SD)).

Items	Total (*n* = 30)	MD (*n* = 16)	SD (*n* = 14)	
	M ± SD	M ± SD	M ± SD	*p*-Value
F/M (*n*)	18/12	9/7	9/5	0.743
Age (yrs)	47.9 ± 12.3	53.1 ± 7.6	43.2 ± 13.7	0.051
BMI (kg·m^−2^)	26.2 ± 4.1	25.8 ± 4.6	26.5 ± 3.8	0.989
WHtR	0.51 ± 0.08	0.51 ± 0.08	0.51 ± 0.07	0.974
BDI	29.4 ± 11.7	18.3 ± 2.4	39.9 ± 8.5	<0.001
pSBP (mmHg)	127.1 ± 10.6	125.6 ± 12.0	128.7 ± 8.7	0.201
pDBP(mmHg)	85.0 ± 10.0	86.5 ± 11.3	86.5 ± 8.8	0.365
cSBP (mmHg)	117.8 ± 10.1	117.2 ± 12.2	118.4 ± 7.5	0.055
cDBP (mmHg)	87.8 ± 10.2	87.8 ± 11.3	87.8 ± 9.1	0.438
PWV (m/s)	7.2 ± 1.1	7.6 ± 1.0	6.8 ± 1.0	0.495
HR (min^−1^)	69.7 ± 9.4	67.9 ± 9.5	72.4 ± 9.1	0.908
RMSSD (ms)	33.7 ± 18.6	33.7 ± 21.2	33.7 ± 15.1	0.510
SDNN (ms)	38.0 ± 18.2	35.6 ± 18.9	41.5 ± 17.6	0.899

*p*-value indicates differences between mildly depressed (MD) and severely depressed (SD) Student’s *t*-test was applied for continuous variables and Chi-square test for categorical variables. F = female, M = Male, BMI = body mass index, WHtR = waist to height ratio, BDI = Beck Depression Inventory, pSBP = peripheral systolic blood pressure, pDBP = peripheral diastolic blood pressure, cSBP = central systolic blood pressure, cDBP = central diastolic blood pressure, PWV = pulse wave velocity, HR = heart rate, RMSSD = root mean square of successive differences between normal heartbeats, SDNN = standard deviation of all normal-to-normal intervals.

**Table 2:** Descriptive and inferential statistics of outcomes.

Outcomes	Total (*n* = 30)				
	Mean Change	*p*-Values (Time)	η^2^	*p*-Values (Time × Group)	η^2^
BMI (kg·m^−2^)	0.14 ± 0.61	0.236	0.05	0.715	0.005
WHtR	−0.002 ± 0.009	0.306	0.037	0.203	0.057
BDI	−20.0 ± 11.6	<0.001	0.871	*p* < 0.001	0.543
pSBP (mmHg)	−4.7 ± 6.8	<0.001	0.322	0.458	0.020
pDBP (mmHg)	−3.5 ± 6.9	0.011	0.209	0.596	0.010
cSBP (mmHg)	−4.8 ± 6.5	<0.001	0.355	0.239	0.049
cDBP (mmHg)	−3.6 ± 6.8	0.008	0.226	0.566	0.012
PWV (m/s)	−0.13 ± 0.23	0.008	0.229	0.340	0.033
HR (min^−1^)	−4.3 ± 8.8	0.015	0.193	0.301	0.038
RMSSD (ms)	12.2 ± 23.9	0.017	0.251	0.236	0.069
SDNN (ms)	10.5 ± 17.8	0.005	0.330	0.108	0.124

Abbreviations: η^2^ = partial eta-square, BMI = body mass index, WHtR = waist to height ratio, BDI = Beck Depression Inventory, pSBP = peripheral systolic blood pressure, pDBP = peripheral diastolic blood pressure, cSBP = central systolic blood pressure, cDBP = central diastolic blood pressure, PWV = pulse wave velocity, HR = heart rate, RMSSD = root mean square of successive differences between normal heartbeats, SDNN = standard deviation of all normal-to-normal intervals.

## Data Availability

The data presented in this study are available on request from the corresponding author.
